# Analysis of c-KIT expression and *KIT* gene mutation in human mucosal melanomas

**DOI:** 10.1038/sj.bjc.6604791

**Published:** 2008-11-18

**Authors:** I Satzger, T Schaefer, U Kuettler, V Broecker, B Voelker, H Ostertag, A Kapp, R Gutzmer

**Affiliations:** 1 Department of Dermatology and Allergology, Skin Cancer Center Hannover, Hannover Medical School, Hannover, Germany; 2 Department of Pathology, Hannover Medical School, Hannover, Germany; 3 Department of Pathology, Klinikum Region Hannover, Hannover, Germany

**Keywords:** melanoma, mucosal, c-KIT, KIT, BRAF

## Abstract

Recent data suggested an increased frequency of *KIT* aberrations in mucosal melanomas, whereas c-KIT in most types of cutaneous melanomas does not appear to be of pathogenetic importance. However, studies investigating the status of the *KIT* gene in larger, well-characterised groups of patients with mucosal melanomas are lacking. We analysed 44 archival specimens of 39 well-characterised patients with mucosal melanomas of different locations. c-KIT protein expression was determined by immunhistochemistry, *KIT* gene mutations were analysed by PCR amplification and DNA sequencing of exons 9, 11, 13, 17 and 18. c-KIT protein expression could be shown in 40 out of 44 (91%) tumours in at least 10% of tumour cells. DNA sequence analysis of the *KIT* was successfully performed in 37 patients. In 6 out of 37 patients (16%) *KIT* mutations were found, five in exon 11 and one in exon 18. The presence of mutations in exon 11 correlated with a significant stronger immunohistochemical expression of c-KIT protein (*P*=0.015). Among the six patients with mutations, in two patients the primary tumour was located in the head/neck region, in three patients in the genitourinary tract and in one patient in the anal/rectal area. In conclusion, *KIT* mutations can be found in a subset of patients with mucosal melanomas irrespective of the location of the primary tumour. Our data encourage therapeutic attempts with tyrosine kinase inhibitors blocking c-KIT in these patients.

Primary mucosal melanomas represent a rare subtype of melanomas that arise from melanocytic cells residing in mucous membranes in various anatomical regions. They account for approximately 1.2% of melanomas (Chang *et al*, 1998) and have mainly been described in the head and neck region, the genitourinary tract and the gastrointestinal tract.

Obviously, sun exposure does not play a role in mucosal melanoma, whereas it is a risk factor in cutaneous melanoma. A recent study described genetic differences between mucosal melanomas and cutaneous melanomas. In this study, four types of melanomas were differentiated, that is, mucosal melanomas, acral melanomas, melanomas on skin with chronic sun damage and melanomas on skin without chronic sun damage. Melanomas arising from skin without chronic sun damage, representing the major group of cutaneous melanomas, were shown to frequently harbour *BRAF* mutations, in particular the *BRAF* V600E mutation. The other melanoma types, including mucosal melanoma, had a high frequency of mutations of the *KIT* gene (Curtin *et al*, 2005, 2006). This finding is in particular intriguing as it may represent a rationale for a targeted therapy with specific tyrosine kinase inhibitors such as c-KIT blockers in mucosal melanomas. To further elucidate the role of c-KIT and *BRAF* in mucosal melanoma, we analysed these two targets in 39 patients with mucosal melanomas treated in our Department.

## Materials and methods

### Patients

Thirty nine patients with mucosal melanomas who were treated in our Department (Skin Cancer Center Hannover) from 1996 to 2007 were retrospectively analysed. A total of 44 archival formalin-fixed and paraffin-embedded tissue samples (35 primary melanomas, 4 lymph node metastases, 2 skin metastases and 3 local recurrences) were accessible for analysis in this study.

### Immunohistopathologic evaluation of c-KIT expression

Highly sensitive immunohistochemistry for c-KIT with a murine monoclonal antibody (clone p145, dilution 1 : 100, DakoCytomation, Hamburg, Germany) was performed as described earlier (Satzger *et al*, 2008) and immunohistochemical stainings were evaluated semiquantitatively. The numbers of positively labelled melanoma cells were scored as follows: 0 for less than 10% positive cells, 1 (+) for 10–25% positive cells, 2 (++) for 26–50% positive cells, 3 (+++) for 51–75% and 4 (++++) for 76–100% positive cells. We did not differentiate between cytoplasmatic and membranous staining as we did not observe isolated surface staining and earlier studies showed cytoplasmatic staining along with membranous staining in melanoma cells positive for c-KIT (Pereira *et al*, 2005; Giehl *et al*, 2007; Rivera *et al*, 2008).

### Mutational analysis of KIT

Tumour cells were isolated from paraffin-embedded tissue (either primary tumour, lymph node metastases, skin metastases or local recurrences), if necessary by micrographic dissection using the PALM Laser-MicroBeam System (PALM Wolfratshausen, Germany). DNA extraction was performed with the DNA extraction kit from Qiagen (Hilden, Germany) following the instructions of the manufacturer. Exons 9, 11, 13, 17, 18 of *KIT* were amplified by LightCycler PCR using specific primers as described in the literature (Tarn *et al*, 2005; Curtin *et al*, 2006). Polymerase chain reaction products were DNA sequenced using an ABI Prism 3700 DNA Analyzer (SeqLab, Göttingen, Germany).

### Mutational analysis of BRAF

To detect the *BRAF* V600E mutation a LightCycler fluorescence resonance energy transfer (FRET) assay with two fluorescent hybridisation probes was performed as described earlier (Hay *et al*, 2007). Real-time PCR was performed by using LightCycler FastStart DNA Master HybProbe (Roche Diagnostics GmbH, Mannheim, Germany). Post amplification fluorescent melting curve analysis was performed by gradual heating of the samples at a rate of 0.2°C per second from 45 to 95°C. Fluorescent melting peaks were determined by plotting of the negative derivative of fluorescence with respect to temperature.

All PCR products that showed deviation from the Wt (wild-type) genomic DNA melting peak as well as from the positive control samples were confirmed by direct sequencing of exon 15 of the *BRAF* gene (SequiServe, Vaterstetten, Germany).

### Statistical analyses

The software SPSS 13.0 was used for statistical analyses. Kaplan–Meier tests and unpaired *t*-tests were performed.

## Results

**Figure 1:**
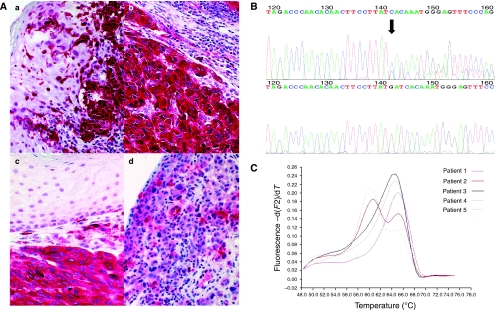
(**A**) Immunhistochemical staining of c-KIT in primary mucosal melanoma showing strong positive membranous and cytoplasmatic labelling (++++) of primary melanoma (**a**), lymph node metastases (**b**) and skin metastases (**c**) of patient 27 (magnification × 100) and, weak and inhomogeneous labelling (+) of primary melanoma (**d**) of patient 18 (magnification × 100). (**B**) KIT genotyping shows a deletion 579 in exon 11 (patient 24). The upper curve depicts the KIT exon 11 forward sequence and the lower curve depicts the wild-type sequence. (**C**) Melting curve analysis of five patients with mucosal melanomas. Melting curve peaks at 64.7°C from BRAF wild type (Patient nos.1, 3 and 4, corresponding to patients 1, 3 and 4 in Table 1) and melting curve peak at 60.6°C from BRAF V600E heterozygous mutation (cases 2 and 5, corresponding to cases 16 and 38 in Table 1).

c-KIT expression was determined in 44 tissue samples, 35 primary mucosal melanomas, 4 lymph node metastases, 2 skin metastases and 3 local recurrences (Table 1). In all, 31 out of 35 (89%) primaries, 4 out of 4 (100%) lymph node metastases, 2 out of 2 (100%) skin metastases and 3 out of 3 (100%) local recurrences were positive for c-KIT (Figure 1A). c-KIT protein expression could be detected in 18 out of 18 (100%) mucosal melanomas located in the head/neck area, 7 out of 8 (86%) in the anal/rectal tract, 8 out of 11 (73%) in the genitourinary tract and 2 out of 2 (100%) in other locations. Score 1+ was determined in 8 out of 44 samples (18%), score 2+ in 7 out of 44 (16%) samples, score 3+ in 9 out of 44 (20%) samples and score 4+ in 16 out of 44 samples (36%). The degree of the c-KIT expression did not correlate with disease-free survival (*P*=0.44) and overall survival (*P*=0.82) by Kaplan–Meier analysis during a follow-up of 31.1 months (mean) and 21.1 months (median, minimum 2.8 months, maximum 151.6 months). During that follow-up period, 20 patients suffered from melanoma recurrence and 14 patients died due to melanoma metastases.

Alterations in the *KIT* gene were observed in 6 out of 37 (16%) patients, five in exon 11 and one in exon 18 (Figure 1B). Two patients suffered from mucosal melanomas of the head/neck region, three patients from mucosal melanomas located in the genitourinary tract and one patient from mucosal melanoma located in the anal/rectal tract. In one patient (case 27) the *KIT* mutation could be detected both in lymph node metastases and in skin metastases. Among the five tumours with *KIT* gene mutation of exon 11, four (80%) tumours showed strong (++++) and one showed (20%) high (+++) c-KIT protein expression (Figure 1A). In contrast, tumours without mutation in exon 11 had significantly lower c-KIT expression (3 out of 32 negative, 7 out of 32 (+), 7 out of 32 (++), 8 out of 32 (+++), 7 out of 32 (++++), *P*=0.015).

In 3 out of 27 (11%) mucosal melanomas (located in head/neck area, pleura and conjunctiva, respectively) the *BRAF* V600E mutation could be detected (Table 1, Figure 1C).

## Discussion

A recent report showed a possible role of c-KIT in subsets of melanoma, in particular, mucosal melanomas (21% *KIT* mutations, 61% c-KIT overexpression), acral cutaneous melanomas (11% *KIT* mutations, 75% c-KIT overexpression) and cutaneous melanomas on skin with chronic sun damage (17% *KIT* mutations, 100% c-KIT overexpression) (Curtin *et al*, 2006). This suggests that c-KIT might be of pathogenetic relevance and therefore a therapeutic target in these subtypes of melanoma. In contrast, *KIT* mutations are rarely found in the major subtype of cutaneous melanoma originating from skin without chronic sun damage (Curtin *et al*, 2006) and unselected cutaneous melanomas (2 out of 100 in Willmore-Payne *et al* (2005); 1 out of 39 in Went *et al* (2004)). Moreover, therapeutic phase II studies with the c-KIT blocker imatinib in unselected melanoma patients without known *KIT* mutation status were disappointing (Ugurel *et al*, 2005; Wyman *et al*, 2006; Becker *et al*, 2007).

The aim of this study was to further elucidate c-KIT alterations in our well-characterised group of patients with mucosal melanomas that might support the role of c-KIT as a new therapeutic target in this subgroup of melanoma. The successful genetic analysis of *KIT* in 37 patients revealed mutations in 6 patients (16%). We could show *KIT* mutations in 2 out of 12 mucosal melanomas from head/neck, 3 out of 11 from the genitourinary tract and 1 out of 8 from the anal/rectal tract. This is consistent with the findings of Antonescu *et al* and Rivera *et al* who detected mutations of the *KIT* gene in 3 out of 20 (15%) and 4 out of 18 (22%) patients with mucosal melanomas of the anal region and oral cavity, respectively (Antonescu *et al*, 2007; Rivera *et al*, 2008). Thus, *KIT* mutations occur in up to 20% of mucosal melanomas irrespective of the location of the primary tumour.

The majority of *KIT* mutations in mucosal melanomas (11 out of 16 tumours in Curtin *et al* (2006), 3 out of 3 in Antonescu *et al* (2007), 4 out of 4 in Rivera *et al* (2008) and 5 out of 6 in our study) were detected in the juxtamembrane region of *KIT* encoded by exons 11 and 13, presumably resulting in the activation of c-KIT.

The L576P und W557R mutations of our patients have already been described both in mucosal melanomas and gastrointestinal stromal tumours (GIST) (Antonescu *et al*, 2004, 2007; Rivera *et al*, 2008). Deletions covering the 579 position (such as in our patient 24) have also been frequently described in GIST (Tarn *et al*, 2005). The K550N mutation found in our patient 2 has, to our knowledge, not been described yet. However, other alterations in the proximal part of exon 11 at codons 550–562 have been reported frequently in GIST (Lasota *et al*, 1999; Longley *et al*, 2001).

Thus, the minor subgroup of patients with mucosal melanomas and activating *KIT* mutations might be susceptible to therapeutic c-KIT blockade. This is supported by findings in GIST, which show *KIT* mutations in 75–80%, and respond significantly better to a therapy with the c-KIT inhibitor imatinib than tumours without *KIT* mutations (Heinrich *et al*, 2003). Therefore, therapeutic c-KIT blockade could be considered for the treatment of patients with mucosal melanomas and an activating *KIT* mutation. This is supported by two case reports published very recently of single patients suffering from metastasising anal melanoma that harboured a *KIT* mutation in exon 11 and exon 13, respectively. These patients were successfully treated with the c-KIT blocker imatinib (Hodi *et al*, 2008; Lutzky *et al*, 2008).

c-KIT protein expression could be observed in 91% of our primary mucosal melanomas, which is similar to the rates reported in other series of primary mucosal melanomas of the anal/rectal tract (12 out of 16 in Chute *et al*, 2006) and oral cavity (16 out of 18 in Rivera *et al*, 2008), respectively. However, Antonescu *et al* (2007) reported lower number (6 out of 26 (23%)) of c-KIT positivity in mucosal melanomas of the anal/rectal tract. In addition, cutaneous melanomas have been reported to express c-KIT by immunohistochemistry in 22.8% (Potti *et al*, 2003) up to 84% (Giehl *et al*, 2007) of the cases. The high range between these studies is being explained with the different qualities of immunohistochemistry. Thus, c-KIT protein expression appears to be in a similar range in mucosal melanomas and cutaneous melanomas. In line with this, Giehl *et al* could find no differences of the c-KIT expression in melanomas with sun exposure compared with melanomas without sun exposure (Giehl *et al*, 2007). In contrast to metastatic melanoma from primary cutaneous melanoma, where the c-KIT expression is decreased (Montone *et al*, 1997; Shen *et al*, 2003), we could observe c-KIT expression in 9 out of 9 metastases of primary mucosal melanoma. Similarly, Antonescu *et al* detected c-KIT reactivity in 6 out of 6 metastases from their anal mucosal melanomas (Antonescu *et al*, 2007). Furthermore, in agreement with earlier studies we could not show a prognostic relevance of the c-KIT protein expression in mucosal melanomas (Chute *et al*, 2006).

We could observe a significant higher immunohistochemical expression of c-KIT in mucosal melanomas that harbour a potentially activating *KIT* mutation as compared with tumours without *KIT* mutation. A similar correlation has also been found for anal melanomas (Antonescu *et al*, 2007) and melanomas of the oral cavity (Rivera *et al*, 2008).

Thus, immunohistochemistry might be a useful tool to screen for patients that are subjected to *KIT* mutation analysis. However, immunohistochemistry might not be sufficient to detect tumours with mutations susceptible for c-KIT blockade, as overexpression of c-KIT can also occur in tumours without mutation. This is illustrated by the report of three patients with metastatic melanoma and strong immunohistochemical c-KIT expression who did not respond to a therapy with the c-KIT blocker imatinib (Alexis *et al*, 2005).

*BRAF* mutations were rarely identified in 3 out of 27 (11%) patients in our series, similarly to other studies with patients suffering from mucosal melanomas who reported 1 out of 17 (Cohen *et al*, 2004) and 0 out of 13 (Edwards *et al*, 2004), respectively, whereas the majority of cutaneous melanomas on skin without chronic sun-induced damage harbour *BRAF* V600E mutations (Curtin *et al*, 2005). It is interesting to note that one of our patients with a *BRAF* mutation suffered from melanoma located in an UV-exposed area of the conjunctiva. In line with this, Gear *et al* (2004) reported *BRAF* mutations in 5 out of 22 patients with conjunctival melanomas.

In conclusion, our study supports the finding that *KIT* mutations presumably activating the tyrosine kinase activity of c-KIT can be found in a subgroup of patients with mucosal melanomas irrespective of the origin of the primary tumour. This encourages clinical studies with c-KIT blockers in patients with mucosal melanomas and appropriate *KIT* mutations. Immunohistochemistry for c-KIT expression might be a useful tool to screen for patients who are subjected to mutational analysis but cannot replace genetic analysis.

## Figures and Tables

**Table 1:** Characterisation of patients, expression of c-KIT by immunohistochemistry (IHC), mutational status of KIT and BRAF in mucosal melanoma, clinical follow-up

**Case**	**Age (years)**	**Gender**	**Location**	**Tissue sample**	**IHC c-KIT**	***KIT* status**	***BRAF* status**	**Follow-up**
1	80	F	Head/Neck	PM	++++	Wt	Wt	Progression in 9 months
2	44	M	Head/Neck	PM	+++	Exon 11, K550N	Wt	Progression in 5 months
3	69	F	Head/Neck	PM	+	NA	Wt	Death due to melanoma in 74 months
				LN	++	Wt	Wt	
4	75	M	Head/Neck	PM	+++	Wt	Wt	Death due to melanoma in 8 months
5	52	M	Head/Neck	PM	+++	Wt	NA	No progression
6	75	M	Head/Neck	PM	+++	Wt	Wt	Progression in 27 months
7	59	M	Head/Neck	PM	++++	NA	NA	Progression in 8 months
				LR	++++	Wt	Wt	
8	77	M	Head/Neck	LR	+	Wt	NA	Progression in 24 months
9	80	F	Head/Neck	PM	++++	Wt	Wt	No progression
10	64	M	Head/Neck	PM	++++	Wt	Wt	No progression
11	68	F	Head/Neck	PM	+++	Wt	NA	Death due to melanoma in 11 months
12	72	M	Head/Neck	PM	++	Wt	Wt	Death due to melanoma in 25 months
13	78	M	Head/Neck	PM	++++	Wt	Wt	Progression in 11 months
14	94	M	Head/Neck	PM	++++	Exon 11, W557R	Wt	Death due to melanoma in 7 months
15	63	F	Head/Neck	PM	++	Wt	Wt	Death due to melanoma in 5 months
16	57	M	Head/Neck	PM	+	Wt	hetero	Death due to melanoma in 6 months
17	59	M	Head/Neck	PM	+++	Wt	Wt	No progression
18	80	F	Head/Neck	PM	+	Wt	Wt	No progression
19	22	F	Genital tract	PM	negative	NA	NA	No progression
20	59	F	Genital tract	PM	+	Wt	NA	Progression in 33 months
21	72	F	Genital tract	PM	++++	Wt	NA	No progression
22	65	F	Genital tract	PM	+++	Wt	Wt	Death due to melanoma in 32 months
23	86	F	Genital tract	PM	negative	Wt	Wt	NA
24	65	F	Genital tract	PM	++++	Exon 11, 579del	Wt	No progression
25	50	F	Genital tract	PM	++	Exon 18, I841V	Wt	No progression
26	81	F	Genital tract	PM	negative	Wt	NA	Death due to melanoma in 37 months
27	76	F	Genital tract	PM	++++	NA	NA	Death due to melanoma in 13 months
				SM	++++	Exon 11, L576P	NA	
				LN	++++	Exon 11, L576P	NA	
28	65	F	Genital tract	LR	+++	Wt	Wt	Death due to melanoma in 11 months
29	74	F	Urinary tract	PM	++++	NA	Wt	Death due to melanoma in 114 months
30	61	M	Anal/rectal	PM	++++	Wt	NA	Death due to melanoma in 51 months
31	65	M	Anal/rectal	PM	negative	Wt	NA	Death due to melanoma in 25 months
32	65	F	Anal/rectal	PM	+++	Wt	Wt	Death due to melanoma in 16 months
33	73	M	Anal/rectal	PM	++	Wt	Wt	Progression in 13 months
				SM	++++	Wt	Wt	
34	66	M	Anal/rectal	PM	+	Wt	Wt	Death due to melanoma in 24 months
35	55	M	Anal/rectal	LN	++	Wt	NA	Progression in 26 months
36	79	F	Anal/rectal	PM	++++	Exon 11, L576P	Wt	Progression in 9 months
37	64	F	Anal/rectal	PM	+	Wt	NA	Distant metastasis at diagnosis
38	52	F	Conjunctiva	LN	+	Wt	hetero	Death due to melanoma in 137 months
39	53	F	Pleura	PM	++	Wt	hetero	Progression in 3 months

hetero=heterozygous, LN=lymph node metastasis; LR=local recurrence, NA=not available, PM=primary melanoma, SM=skin metastasis, Wt=wild-type.

IHC-assessment: negative for less than 10% positive cells, +for 10–25% positive cells, ++ for 25–50% positive cells, +++ for 50–75% and ++++ for 75 to 100% positive cells.
